# Competency assessment tool for laparoscopic suturing: development and reliability evaluation

**DOI:** 10.1007/s00464-019-07077-2

**Published:** 2019-08-26

**Authors:** Wouter M. IJgosse, Erik Leijte, Sandeep Ganni, Jan-Maarten Luursema, Nader K. Francis, Jack J. Jakimowicz, Sanne M. B. I. Botden

**Affiliations:** 1 Department of Surgery, Radboud University Medical Center, Geert Grooteplein zuid 10, 6525 GA Nijmegen, Gelderland The Netherlands; 2 Research and Education, Catharina Hospital, Michelangelolaan 2, 5653 EJ Eindhoven, The Netherlands; 3 Delft University of Technology, Industrial Design Engineering, Medising, Delft, The Netherlands; 4 Department of Surgery, GSL Medical College, Rajahmundry, India; 5 Department of Surgery, Yeovil District Hospital NHS Foundation Trust, Yeovil, UK; 6 Division of Pediatric Surgery, Department of Surgery, Radboudumc - Amalia Children’s Hospital, Nijmegen, The Netherlands; 7 Radboud University Medical Center, PO Box 9101 (960), 6500 HB Nijmegen, The Netherlands

**Keywords:** Training, Evaluation, Laparoscopic suturing, Objective assessment, Simulation

## Abstract

**Background:**

Laparoscopic suturing can be technically challenging and requires extensive training to achieve competency. To date no specific and objective assessment method for laparoscopic suturing and knot tying is available that can guide training and monitor performance in these complex surgical skills. In this study we aimed to develop a laparoscopic suturing competency assessment tool (LS-CAT) and assess its inter-observer reliability.

**Methods:**

We developed a bespoke CAT tool for laparoscopic suturing through a structured, mixed methodology approach, overseen by a steering committee with experience in developing surgical assessment tools. A wide Delphi consultation with over twelve experts in laparoscopic surgery guided the development stages of the tool. Following, subjects with different levels of laparoscopic expertise were included to evaluate this tool, using a simulated laparoscopic suturing task which involved placing of two surgical knots. A research assistant video recorded and anonymised each performance. Two blinded expert surgeons assessed the anonymised videos using the developed LS-CAT. The LS-CAT scores of the two experts were compared to assess the inter-observer reliability. Lastly, we compared the subjects’ LS-CAT performance scores at the beginning and end of their learning curve.

**Results:**

This study evaluated a novel LS-CAT performance tool, comprising of four tasks. Thirty-six complete videos were analysed and evaluated with the LS-CAT, of which the scores demonstrated excellent inter-observer reliability. Cohen’s Kappa analysis revealed good to excellent levels of agreement for almost all tasks of both instrument handling and tissue handling (0.87; 0.77; 0.75; 0.86; 0.85, all with *p *< 0.001). Subjects performed significantly better at the end of their learning curve compared to their first attempt for all LS-CAT items (all with *p *< 0.001).

**Conclusions:**

We developed the LS-CAT, which is a laparoscopic suturing grading matrix, with excellent inter-rater reliability and to discriminate between experience levels. This LS-CAT has a potential for wider use to objectively assess laparoscopic suturing skills.

Over the past two decades, Minimal Invasive Surgery (MIS) has expanded rapidly with more advanced surgical operations now being performed laparoscopically. This often involves carrying out reconstructive procedures which requires the skills of performing laparoscopic suturing [1, 2].

Training for laparoscopic suturing is an integral part of the laparoscopic surgical curriculum [3] and has moved from the operating room to a skills lab setting [4]. Complex surgical skills such as laparoscopic suturing and knot tying are challenging due to the inherent limitations of MIS such as an altered depth perception, two-dimensional vision, ergonomic issues and the small working field [5, 6].

Extensive training, therefore, is required to overcome these limitations and to achieve competency and is often based on the principle of modelling, repetitive practice and formative feedback [7]. Surgical residents are currently more and more restricted in their clinical working hours, reducing their opportunities for gaining practical surgical experience. Therefore, assessment of performance is required not only to ensure competency but to guide and enhance the efficiency of learning [8]. Assessment of laparoscopic suturing is traditionally dependent on subjective evaluation by trainers since objective evaluation has not yet been established.

Several attempts to objectively assess laparoscopic suturing have been reported in literature including the use of virtual reality (VR) simulation, motion-tracking systems or check lists. The application of VR to objectively evaluate laparoscopic suturing skills can be challenging [3]. VR simulators are able to fully assess the trainees, but lack the important haptic feedback, needed for laparoscopic suturing [8]. There are several studies which applied a motion-tracking system to real-time performance [6, 9] to objectively appraise the operative performance of this complex task, but this method is of limited generalisability and external validity. There are various other measurement tools available, but they vary in their objectivity, validity and reliability [10]. Mandel et al. mentioned the importance of immediate and specific feedback during training and suggests the use of task-specific and global checklists for both learning and self-assessment [11].

A competency assessment tool (CAT) is a method to assess laparoscopic performance, by describing specific steps in the process of the specific task and evaluates both the process of performance (instrument use, tissue handling and committed errors) and the quality of the end product. The CAT tool has been successfully applied to approve the quality of training in the English National Training Programme for laparoscopic colorectal surgery [12]. Considering the importance of laparoscopic suturing and its wide application within the practice of MIS, there is a clear need for an objective assessment tool that can reliably appraise the operative performance of such complex technique. We therefore aimed to develop a bespoke CAT for laparoscopic suturing and assess the reliability of the tool by assessing the inter-observer reliability.

## Materials and methods

### Development of competency assessment tool

The development of the laparoscopic suturing CAT (LS-CAT) was performed with a structured, mixed methodology approach and overseen by a steering committee with experience in developing surgical assessment tools and objective assessment of laparoscopic rectal surgery. A wide Delphi consultation with over twelve international experts in laparoscopic suturing guided the development stages of the tool. The steps were standardised and agreed first prior to defining the task areas for assessment with the tool. Based upon an expert consensus, we deconstructed the procedure into a series of constituent steps. The final model of the LS-CAT was adapted from the original CAT for assessing colorectal surgery [12].

Next, we used a semi-structured interview framework allowing the experts freedom to express their thoughts and explore ideas, whilst also enabling the interviewer to ensure the necessary information was covered [13]. Open questions were used to determine what indicators of performance the expert would look for to assess technical performance of laparoscopic suturing. Additionally, for each task area, two video clips were prepared for the expert to reflect upon the technical performance displayed. A research assistant transcribed the interviews verbatim and analysed them using qualitative methods. After coding and grouping of the statements and until thematic saturation was achieved, the thematic analysis was performed. We collated descriptors of poor and proficient performance from the transcripts and triangulated them into the specific procedural tasks to which they applied to generate the assessment metrics for the draft tool.

The draft of the LS-CAT consisted of four agreed task areas, reflecting steps of the procedure described in the expert consensus. Based on the interviews and error analysis, we developed objective descriptors for each task and refined them through discussions amongst the steering group. To describe the quality of technical performance for each domain (four) for each task area (two) a four-point ordinal scale was used, where a lower score indicates a more proficient technical performance and a high score (four) a poor performance. A total LS-CAT score of eight indicates a perfect and proficient performance, because one point was scored on both items in each task, without errors during the performance.

### Tool testing

#### Training setup

Training took place at the Radboud University Medical Center, Nijmegen. During the first training session, a research assistant was available to instruct subjects prior to conducting the laparoscopic suturing tasks. The research assistant video recorded and anonymised each performance but was not involved in the LS-CAT scoring process. Each participant performed the suturing tasks multiple times to train along a learning curve.

The LS-CAT was evaluated using the following suturing task:

- A standard suturing task. The participant had to place two surgical knots on a suturing pad in a horizontal plane (double wind followed by two single winds to create a secure surgeon’s knot) with a standard length of 20-cm thread. If the thread of a suture was too short to reuse after being cut by the research assistant, a new suture would be placed on the suture pad.

### Training subjects

Subjects were divided into three groups based on their self-reported laparoscopic experience: (1) novices were subjects without clinical experience but with understanding of the concept of laparoscopy such as medical interns and first-year residents, (2) intermediates with more than ten basic laparoscopic procedures performed but less than twenty advanced laparoscopic procedures and (3) experts with more than twenty advanced laparoscopic procedures performed, therefore consisting of residential surgeons in staff. Because the novices were training on their learning curve, the videos of the end of the learning curve were used as a fourth group.

### Protocol

All participants signed an informed consent for the video recording of their task performances prior to the start of the training. When all participants finished the training, we analysed 36 videos from the bulk of all participants’ performances, after which two blinded expert surgeons completed the LS-CAT independently of each another. Both experts had experience using the original CAT tool [14], but had not used the adapted version for laparoscopic suturing before. Participation was on voluntary basis and subjects received no compensation. No IRB approval was needed for this study.

### Equipment

**Fig. 1:**
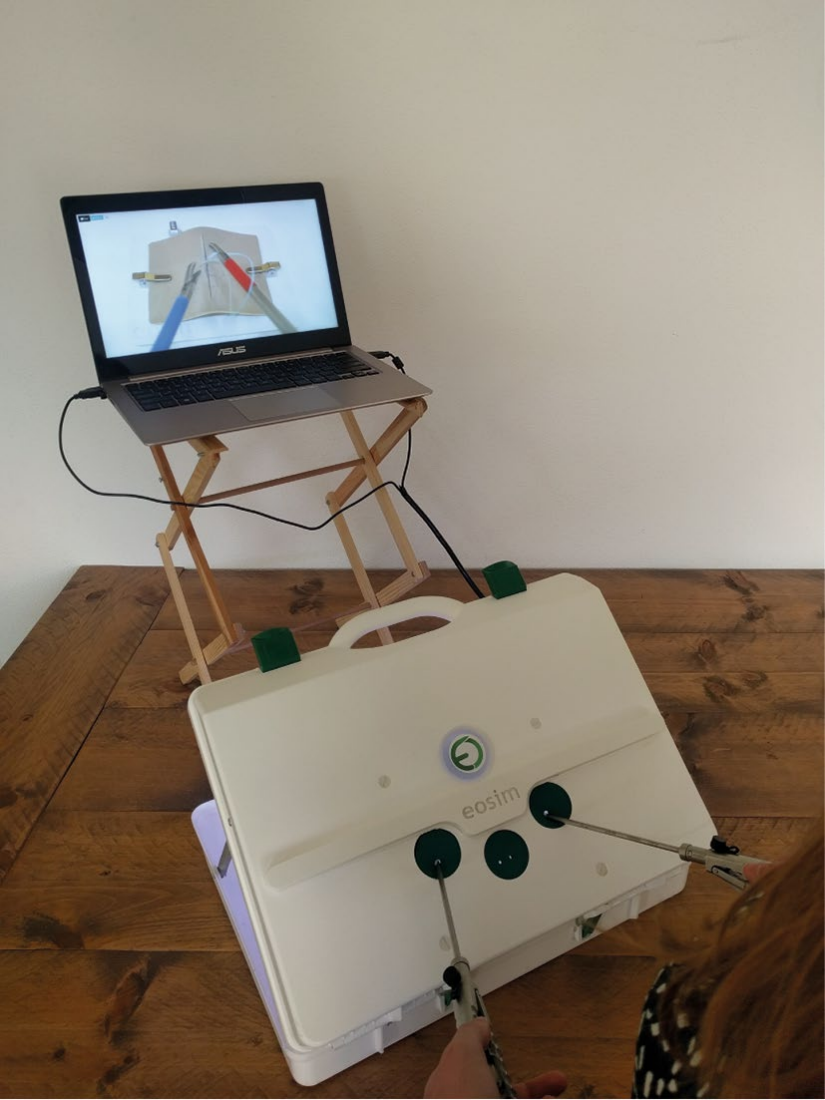
The eoSim-augmented reality laparoscopic simulator interface

The eoSim-augmented reality laparoscopic simulator by eoSurgical Ltd., Edinburgh, Scotland, United Kingdom, was used in this study, in a standard setup (Fig. 1). This setup consisted of the eoSim laparoscopic case with an internal-mounted high-definition camera and standard supplied equipment that consists of laparoscopic instruments, needle holders, a suturing pad, a thread transfer platform and a box with standard exercise equipment, combined with a 15-inch laptop with the required specification as recommended by eoSurgical and the eoSurgical SurgTrac software installed. The tracking camera, that is mounted in the case, was connected to the laptop via USB 2.0 and used to record each performance of the participant. For every participant, the height of the laptop screen was adjusted to the proper height with the laparoscopic box being placed on a standard height table. Participants used a 30-mm curved needle braided thread suture to perform the task.

### Statistical analysis

All statistical analyses were performed with IBM’s SPSS statistics v.25 package. First the total scores for instrument handling, tissue handling and the amount of errors were calculated. Following, the inter-observer reliability was assessed by using Cohen’s Kappa analysis for the task scores of instrument handling and tissue handling. A κ > 0.75 was considered as an excellent agreement [15]. The inter-observer reliability for the calculated total scores between the two observers was assessed using the Pearson correlation, on a 2-tailed significance level of *p *< 0.01. An *r *≥ 0.8 was considered a high correlation [16]. Lastly, the performance scores at the beginning and end of the learning curve within the novice group were compared using the Mann–Whitney *U* test. This process was conducted by three independent researchers who were not involved in the scoring process using the filled in LS-CAT forms of the observers (EL calculated the total scores, SMBI conducted the statistical analyses, WMIJ repeated both processes as a final check).

## Results

### Development of competency assessment tool

**Fig. 2:**
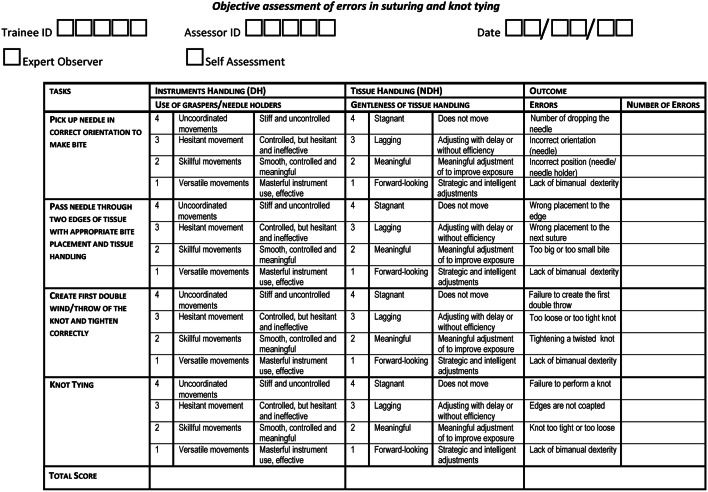
The CAT form for laparoscopic suturing

The final LS-CAT is presented in Fig. 2. Two vertical columns represent task areas, and four horizontal rows represent the performance domains: giving a total of eight separate items which are scored on a scale of 1–4, where a lower score indicates a more proficient technical performance and a total score of eight indicates a perfect and proficient performance. The third column represents the amount of errors which is scored on four domains for each task resulting in 16 separate items.

Four tasks were agreed on and defined from the consensus document for assessment with the tool: (1) pickup needle in correct orientation to make bite; (2) pass needle through two edges of tissue with appropriate bite placement and tissue handling; (3) create first double wind/throw of the knot and tighten correctly and (4) knot tying.

### Reliability

All participants were able to finish the suturing task. In total, 36 videos of eighteen participants were randomly collected and were scored independently by the two objective observers (observer A and B). Of these participants, seventeen were novices and one was an expert. Mean scores for each separate item are presented in Table 1. Cohen’s Kappa analysis revealed good to excellent inter-rater agreement scores for almost all tasks of instrument handling and tissue handling (0.87; 0.77; 0.75; 0.86; 0.85, all with *p *< 0.001, Table 2). The LS-CAT total scores demonstrated excellent inter-observer reliability for instrument handling (*r *= 0.98, *p *< 0.001), tissue handling (*r *= 0.86, *p *< 0.001), errors (*r *= 0.99, *p *< 0.001) and the total assessment score (*r *= 0.98, *p *< 0.001). An overview with more detail is presented in Table 3.

**Table 1:** Scores of the separate items on the LS-CAT. The values are stated in means and standard deviations

	Instrument handling		Tissue handling		Errors	
	A	B	A	B	A	B
Pickup needle in correct orientation	2.7 (0.8)	2.6 (0.8)	2.4 (0.6)	2.3 (0.7)	0.9 (2.0)	0.9 (2.3)
Pass needle through edges of tissue	2.5 (0.7)	2.5 (0.8)	2.3 (0.7)	2.3 (0.8)	0.5 (0.9)	0.5 (0.8)
Create first double throw	2.3 (0.8)	2.4 (0.9)	2.3 (0.7)	2.3 (0.6)	0.2 (0.4)	0.1 (0.3)
Knot tying	2.5 (0.9)	2.4 (0.8)	2.4 (0.7)	2.3 (0.7)	0.5 (0.6)	0.4 (0.5)
Total score	10.0 (2.8)	9.9 (3.0)	9.4 (2.3)	9.1 (2.4)	2.1 (3.2)	1.9 (3.2)

*A* observer A, *B* observer B

**Table 2:** Inter-rater agreement for the categorical variables calculated with Cohen’s Kappa

	Instruments handling		Tissue handling	
	κ	*p*	κ	*p*
Pickup needle in correct orientation	0.87	< 0.001	0.86	< 0.001
Pass needle through edges of tissue	0.77	< 0.001	0.51	< 0.001
Create first double throw	0.73	< 0.001	0.85	< 0.001
Knot tying	0.75	< 0.001	0.73	< 0.001

**Table 3:** Correlations between the total scores of the items

	Observer A	Observer B	*r*	*p*
Instrument handling	10.0 (2.8)	9.9 (3.0)	0.98	< 0.001
Tissue handling	9.4 (2.3)	9.1 (2.4)	0.86	< 0.001
Total score	19.4 (4.9)	19.0 (5.2)	0.96	< 0.001
Total errors	2.1 (3.2)	1.9 (3.2)	0.99	< 0.001
Total assessment score	21.4 (7.1)	20.9 (7.5)	0.98	< 0.001

This is calculated with Pearson correlation, on a 2-tailed significance level of *p *< 0.01

### Performance scores

Within the novice group, subjects performed significantly better at the end of their learning curve compared to their first attempt for all items on the LS-CAT as assessed by both observers. Overall scores are significant for all tasks: instrument handling (*p *< 0.001); tissue handling (*p *< 0.001); pickup needle in correct orientation (*p *< 0.001); pass needle through edges of tissue (*p *< 0.001); create first double throw (*p *< 0.001); knot tying (*p *< 0.001); total amount of errors (*p *< 0.001) and the total assessment score (*p *< 0.001). A full overview of subjects’ mean scores and statistics by observer A and B is presented in Table 4.

**Table 4:** Score comparisons of the first attempt and the last attempt of the separate LS-CAT items as assessed by the Mann–Whitney *U* test

	Observer A				Observer B			
	Mean rank				Mean rank			
	First attempt	Last attempt	*U*	*p*	First attempt	Last attempt	*U*	*p*
Instrument handling	27.06	9.94	8.00	< 0.001	27.19	9.81	5.50	< 0.001
Tissue handling	26.97	10.03	9.50	< 0.001	26.17	10.83	24.00	< 0.001
Pickup needle in correct orientation	27.31	9.69	3.50	< 0.001	27.00	10.00	9.00	< 0.001
Pass needle through edges of tissue	25.64	11.36	33.50	< 0.001	25.58	11.42	34.50	< 0.001
Create first double throw	25.94	11.06	28.00	< 0.001	26.06	10.94	26.00	< 0.001
Knot tying	26.17	10.83	24.00	< 0.001	25.53	11.47	35.50	< 0.001
Total errors	25.94	11.06	28.00	< 0.001	25.19	11.81	41.50	< 0.001
Total score	27.31	9.69	3.50	< 0.001	27.17	9.83	6.00	< 0.001

## Discussion

Laparoscopic suturing is considered as an essential skill that is required in advanced MIS techniques. Currently, there are no reliable tools that are widely used, to objectively appraise performance in this advanced technique. This is required to influence and promote training and ascertain competency. Mandel et al. already suggested the incorporation of task-specific checklist, which has been incorporated in the CAT method with success [11]. The incorporation of this checklist was even accurate for self-assessment [14], which is an important finding, because the usability for self-assessment reduces costs and workload for expert instructors [14, 17].

The original concept of CAT has been proven successful to reliably assess technical performance [12]. Based on the method used for the original CAT development, we developed a bespoke laparoscopic suturing competency assessment tool (LS-CAT) that describes and evaluates agreed specific steps in laparoscopic suturing. It evaluates both the process of performance (instrument use, tissue handling and committed errors) and the quality of the end product. Prior to using this new tool in surgical training, multiple criteria must be met, including reliability evidence [4, 18, 19]. This study demonstrated excellent inter-observer reliability for all variables in the adapted CAT form for laparoscopic suturing. Furthermore, a significant difference in performance was found for subject’ scores at the beginning and end of their learning curve, indicating the ability of the LS-CAT to discriminate between experience levels within the learning curve.

In the clinical setting, skills are often assessed by experts using the Objective Structured Assessment of Technical Skills (OSATS) form based on the overall performance [14, 20, 21]. However, OSATS do not seem to provide any formative information on the separate skills that still needs to be improved or already is sufficient, which the CAT form does. There is also no clear demonstrated correlation between the OSATS score and outcome of the specific procedure that the resident or surgeon has performed [22], furthermore the trainee does not know which specific skills have to be improved. The scoring of tools like OSATS and its derivatives like the Global Evaluative Assessment of Robotic Skills (GEARS) or generic Global Operative Assessment of Laparoscopic Skills (GOALS) are not specifically designed to provide the information on the separate skills that are being trained.

Other instruments such as a General Rating Scale (GRS) are considered a fair measurement tool, because of the adding of some more specific qualitative assessment parameters (rated on a five-point scale). When using video-recorded performances, this could enhance the objectivity in the ratings of both the OSATS and the GRS; however, these are still not as task specific as the CAT form. Another assessment method often used for surgical skills training (outside the clinical setting) is motion tracking, which is a highly objective measurement tool used in virtual and augmented reality, and the validity has been proven for numerous systems [19, 23]. However, the quality of the overall task performance might not be assessed sufficiently, because the parameters used are often abstract and not translated to the actual performance of the procedure. Parameters such as ‘path length’ or ‘economy of motion’ and ‘time’ are used, which are not informative of the outcome of the task [24]. These parameters might give an insight in the expertise level of the trainee, but they do not provide information on the accuracy of the task or the final product to indicate competency. Furthermore, a motion-tracking system seems to be limited to research centres with available resources, which limits its wider use. The mentioned shortcomings of these assessment methods are not present in the LS-CAT and it requires very little resources and can be generalisable in the assessment and training of laparoscopic suturing skills. Therefore, we think it has the potential as an objective performance assessment for laparoscopic suturing.

Another method for assessment along this model is the Crowd-Sourced Assessment of Technical Skills (C-SATS), which is a type of video assessment performed by large numbers of anonymous online raters [10]. These raters are self-selected from broad sections of the public, thus not every rater may have a medical background. Multiple studies have shown that the inter-observer reliability of a large group of non-expert observers was even better than a smaller group of expert observers for the assessment of surgical performance [25–27] which suggest this method could be used as an assessment tool in surgical technical skills education. The combination of C-SATS with the CAT method could be a powerful mix in terms of time management and cost effectiveness. Both the potential of C-SATS and the usability for self-assessment of the (LS-) CAT form need to be researched in future studies, to fully understand their potential benefits to provide a directive and focused assessment for laparoscopic suturing.

A limitation of this study is that the tool was designed to facilitate categorical qualitative appraisal of skill areas within a series of tasks. Whilst this makes it an effective adjunct to breakdown the task for delivery of constructive feedback on performance, there are certain assumptions that may impact upon its use for summative assessment. There is an assumption that performance in each skill domain and each task is of equal importance (weight) to the overall performance of the procedure. Additionally, the assessment metrics used for the tool were defined by the authors in discussion with experts; however, there may be aspects of performance that were not identified and thus are not evaluated in the current tool. Therefore, other studies are required to validate the tool and clarify its role within the training curriculum for laparoscopic surgery.

## Conclusion

We developed the LS-CAT, which is a laparoscopic suturing grading matrix to objectively assess the technical performance of laparoscopic suturing, with an excellent inter-rater reliability and the ability to discriminate between experience levels within the learning curve. Although the LS-CAT satisfies many of the requirements of a useful assessment tool with potential application for summative assessment and guide training in this task, further validation studies are required.
